# Supplementary material for: Refractory Hypotension in a Late-Onset Mitochondrial Encephalomyopathy, Lactic Acidosis, and Stroke-like Episodes (MELAS) Male with m.3243 A>G Mutation: A Case Report
Source: Brain Sci. 2023 Jul 17;13(7):1080. doi: 10.3390/brainsci13071080 (PMC10377322; doi:10.3390/brainsci13071080)
Supplement: Supplementary file 1 [file brainsci-13-01080-s001.zip › brainsci-2461240-supplementary.pdf]

Supplementary Material

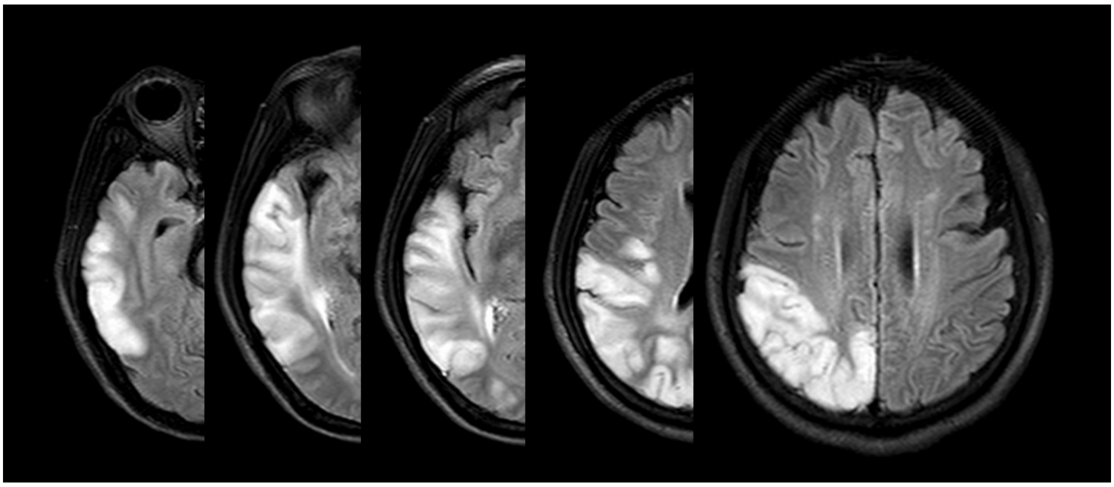

Figure S1. The patient experienced his first stroke-like episode two years ago, prior to the current hospitalization, and was diagnosed with right parieto-occipital temporal lobe cerebral infarction. Brain diffusion-weighted image (DWI) shows right parieto-occipital temporal lobe infarction lesion.

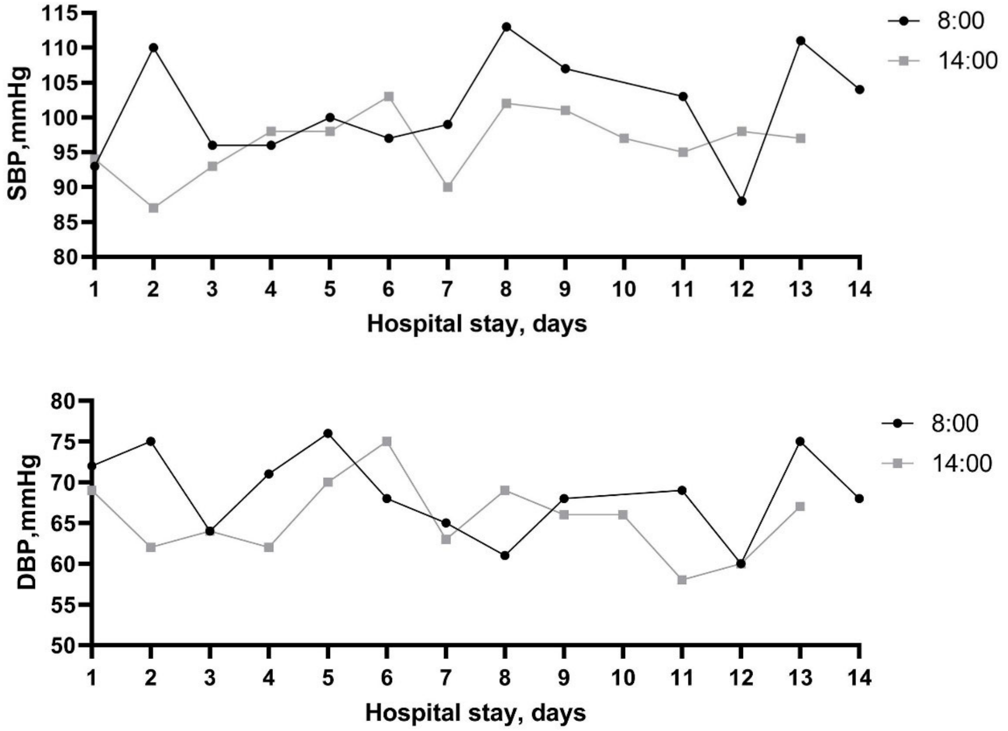

Figure S2. Changes of blood pressure levels during the first time of hospitalization.

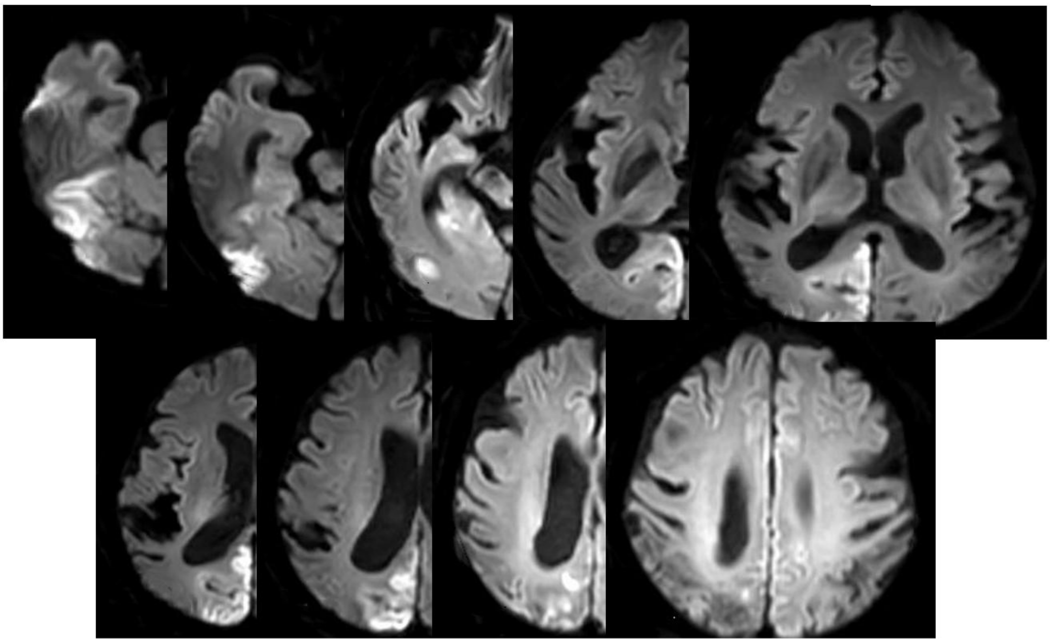

Figure S3. MRI shows multiple acute infarct-like lesions in the right parieto-occipital lobe and bilateral temporal gyrus.
